# Study of a Compression-Molding Process for Ultraviolet Light-Emitting Diode Exposure Systems via Finite-Element Analysis

**DOI:** 10.3390/s17061392

**Published:** 2017-06-15

**Authors:** Kuo-Tsai Wu, Sheng-Jye Hwang, Huei-Huang Lee

**Affiliations:** 1 Department of Mechanical Engineering, National Cheng Kung University, No. 1, University Road, Tainan 701, Taiwan; n18991194@mail.ncku.edu.tw; 2 Department of Engineering Science, National Cheng Kung University, No. 1, University Road, Tainan 701, Taiwan; hhlee@mail.ncku.edu.tw

**Keywords:** wafer level lens, finite element method (FEM), compression molding

## Abstract

Although wafer-level camera lenses are a very promising technology, problems such as warpage with time and non-uniform thickness of products still exist. In this study, finite element simulation was performed to simulate the compression molding process for acquiring the pressure distribution on the product on completion of the process and predicting the deformation with respect to the pressure distribution. Results show that the single-gate compression molding process significantly increases the pressure at the center of the product, whereas the multi-gate compressing molding process can effectively distribute the pressure. This study evaluated the non-uniform thickness of product and changes in the process parameters through computer simulations, which could help to improve the compression molding process.

## 1. Introduction

The first nanoimprint technology was developed by Chou and Krauss [1] using a process called thermoplastic nanoimprint lithography (NIL), which successfully transferred a 10-nm line pattern. This was a major breakthrough in nanometer-scale processing technology and has since been used for producing optical components, medical chips, semiconductors, and solar cells.

There are many types of NILs, of which the most important and popular are thermoplastic NIL, soft lithography, and photo NIL (P-NIL). Soft lithography, also known as micro-contact printing, was developed and introduced by Whitesides et al. [2]. It involves a collection of techniques based on printing, molding, and embossing with an elastomeric stamp. Ultraviolet NIL (UV-NIL) was developed by Sreenivasan and Willson [3], who presented a design for the orientation stages used for high-resolution imprint lithography machines.

The current UV-NIL equipment that is commercially available is made for the micro-imprinting process of wafer-level camera lenses; however, this technology has several disadvantages, such as low yield rate, low throughput, and high operation complexity. UV-NIL is also promising for the high-throughput production of patterns owing to its compatibility with room-temperature processing in contrast to thermal NIL [4]. Among the various hot-embossing processes, injection-compression molding (ICM) is one of the lowest cost and highest throughput techniques [5,6,7]. Nagato [8] demonstrated the fabrication of replica molds for UV-NIL by ICM, which is a proven technique for molding optical disks, such as compact disks (CDs), digital versatile disks (DVDs), and Blu-ray disks (BDs) with low cost and high throughput [9,10]. However, this process has several disadvantages, such as warpage, material shrinkage, and unevenness.

Chen [11] developed a simulation for the ICM filling stage and further proposed simulations of the ICM process based on the Leonov viscoelastic fluid model [12]. Chang [13] used a body-fitted finite-element method (FEM) to simulate the ICM process and modified the process parameters to understand the effects of various processing conditions, such as the compression velocity and the compression gap. Li [14] used 3D modeling and the coupled calculation method for flow, temperature, and pressure to simulate the ICM process for an irregular geometric transparent plastic panel. This approach was used to accurately calculate the shrinkage rate in the depth of the panel and predict its warpage. To achieve more rigorous modeling and simulation, Dwiyantoro [15] presented a numerical study using a moving grid for the simulation of the melt in the ICM process. Ho [16] and Cao [17] proposed three-dimensional numerical analysis systems using a stabilized FEM and an arbitrary Lagrangian–Eulerian (ALE) method for more rigorous modeling and simulation of the three-dimensional geometry of the ICM process.

However, in the compression molding process, which is limited by the material’s viscosity, it is only possible to use pinpoint gates for filling. Thus, in the compression process, the UV-curable resin does not uniformly coat the substrate, causing an uneven pressure distribution that induces a non-uniform thickness, which is a serious issue in this process. In addition to the process parameters discussed in previous studies (e.g., compression velocity and compression gap), in this research, we examine the effects of different gate types on the non-uniform thickness problem. The mold–flow and structural analyses are used to predict the non-uniform thickness with varying gate types for optimizing the thickness uniformity.

## 2. Materials and Methods

**Figure 1:**
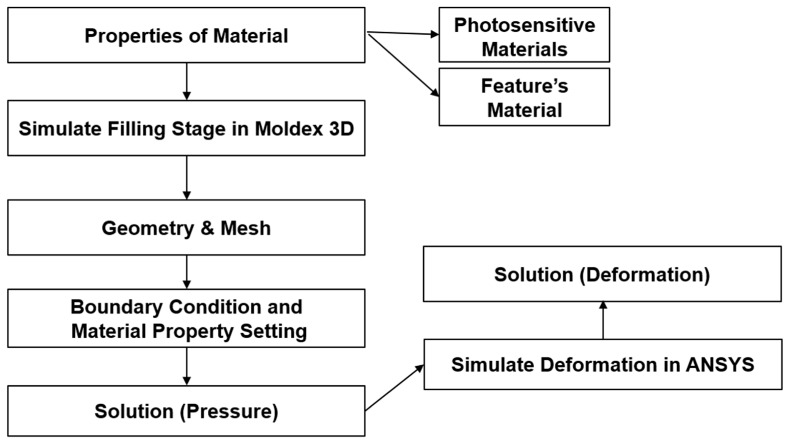
A flow chart showing finite-element analysis.

We used the Moldex3D software to simulate the compression-molding process, followed by the use of the simulation result for stress distribution as the loading into ANSYS for structural analysis, as show in Figure 1. This process was used to simulate the deformation of soft polymer dies, which is indicative of the non-uniform thickness of the product.

### 2.1. Problem Description

The UV-curable resin fills a soft substrate on the mold, with a force pushing upward on the bottom of the substrate, causing the compound to fill the mold. To obtain uniform filling, the bottom has three pins to detect the resultant force, which cannot estimate forces above the rated pressure. When the compression force reaches the rated pressure, the process pauses and allows the UV-curable resin to spread until the pins detect a decrease in the reaction force. It will then continue to compress the mold. Even with this mechanism, there will still be an uneven pressure distribution, and thus, the product has a non-uniform thickness after the compression-molding process. We focus our attention on this issue.

**Figure 2:**
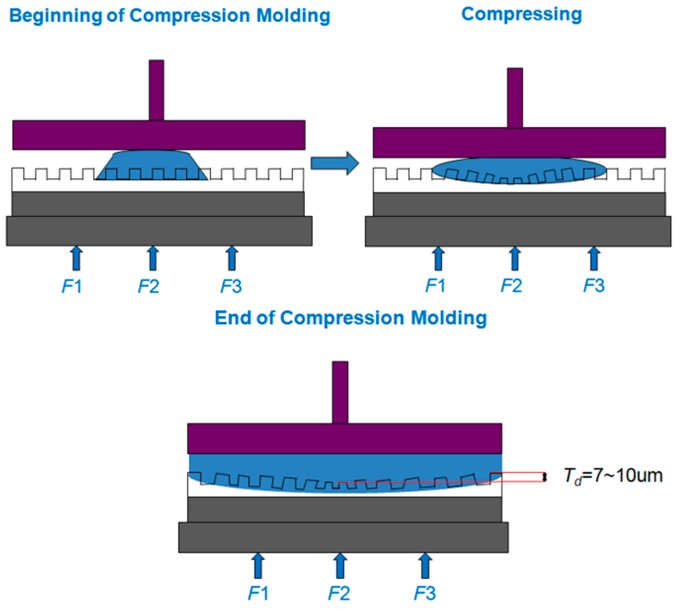
Schematic of the process for filling ultraviolet (UV)-curable resin in a soft mold, resulting in non-uniform thickness.

As shown in Figure 2, the center of the mold will experience more pressure than the other regions of the mold because the UV-curable resin will have greater deformation at the center than on the outer edges, indicating that the center and the periphery have a significant difference in thickness.

**Figure 3:**
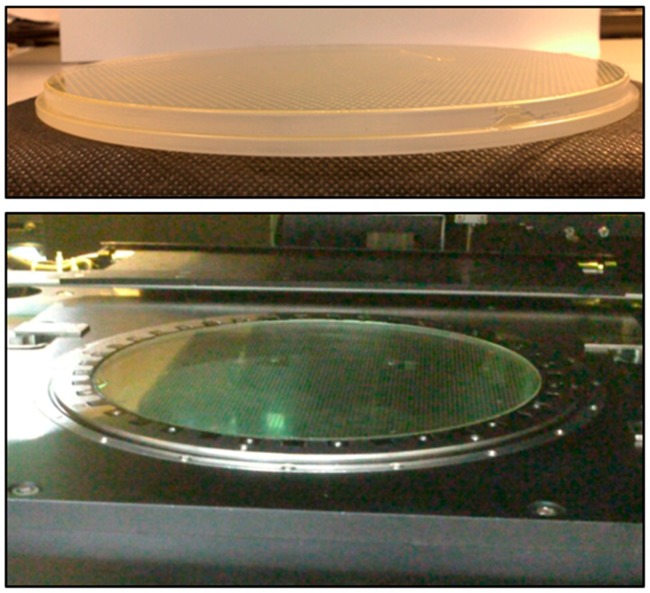
Soft polymer dies.

**Figure 4:**
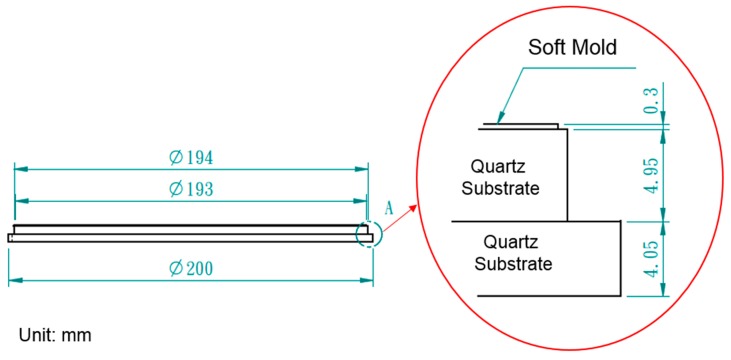
Dimensions of the soft polymer dies.

The geometric model of the imprint machine is based on the information provided by Contrel Technology Co., Ltd. (Tainan, Taiwan). The cavity side of mold is shown in Figure 3, and its dimensions are shown in Figure 4. There are two layers of quartz substrates with thicknesses of 4.95 and 4.05 mm, respectively, and a 0.3-mm-thick layer of the soft polymer.

**Figure 5:**
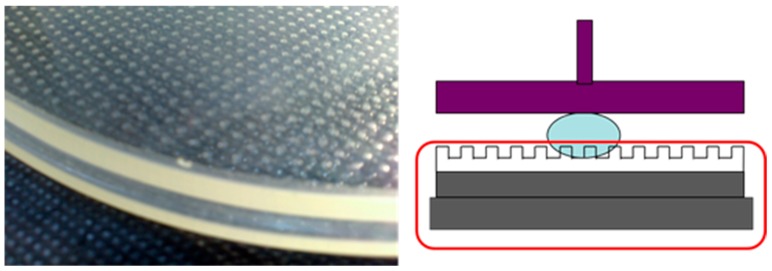
Micro-convex features on the soft polymer dies.

There are many micro-convex features on the soft polymer dies, as shown in Figure 5.

### 2.2. Establishing the Simulation Models

**Figure 6:**
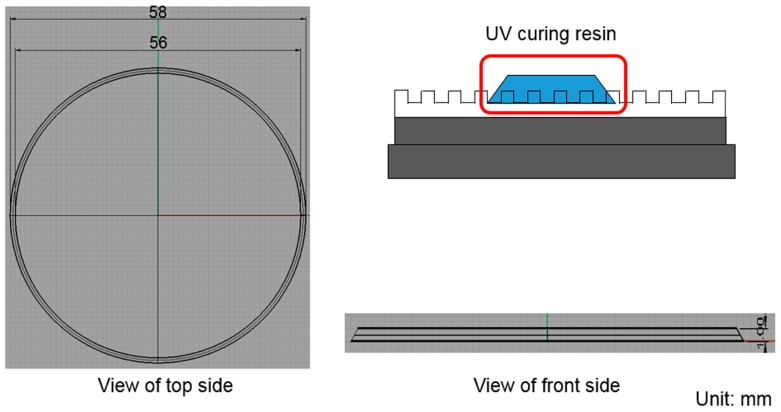
The filled UV-curable resin was modeled as a flattened cone to simulate the compression process with a contact angle of 45°.

The UV-curable resin was modeled as a complete filling shape (shape of a flat-top cone) in the simulation to observe the changes in the shape throughout the compression process. Different filling shapes with contact angles of 45° and 135° were modeled for the UV-curable resin in the initial simulation. The results show that there was no significant difference between these models; therefore, a contact angle of 45° was used to simulate the filling shape. As shown in Figure 6, the flat-top cone had an upper diameter of 56 mm, a lower diameter of 58 mm, and a height of 1 mm.

### 2.3. Material Properties and Process Conditions

**Figure 7:**
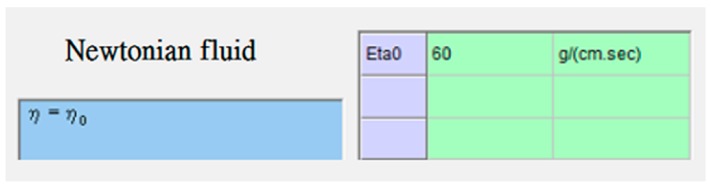
Viscosity model (UV-curable resin) in Moldex3D: Newtonian fluid.

**Figure 8:**
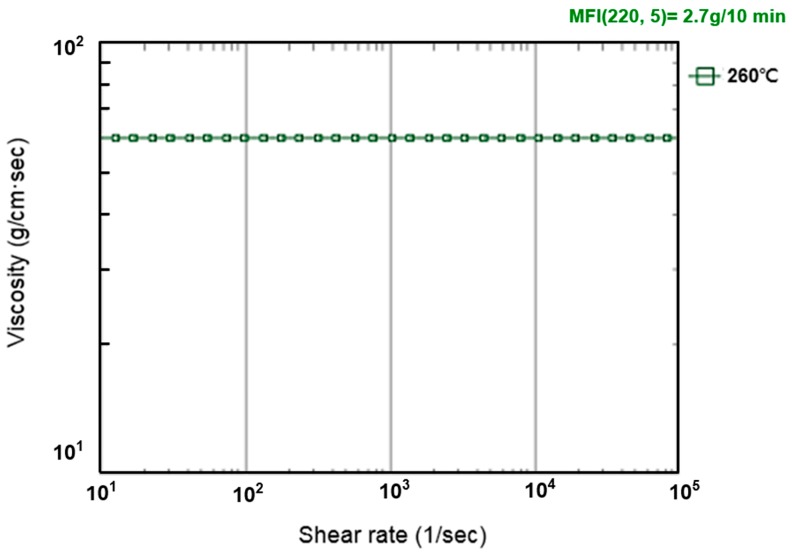
Viscosity curve in Moldex3D of the UV-curable resin.

**Figure 9:**
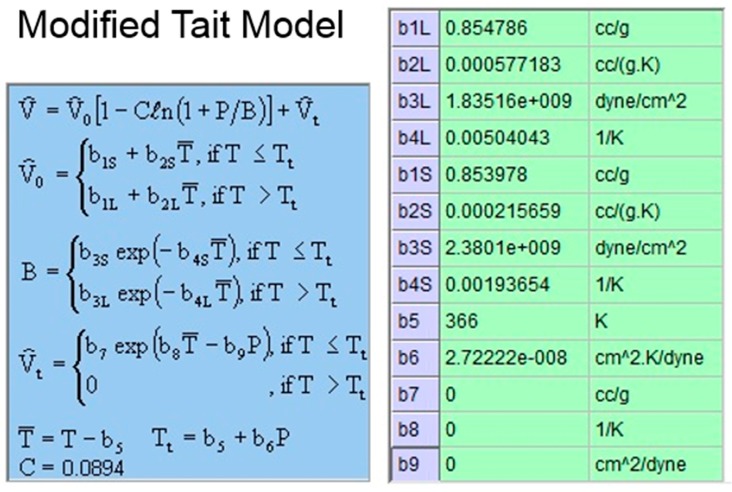
Specific volume model in Moldex3D of the UV-curable resin.

**Figure 10:**
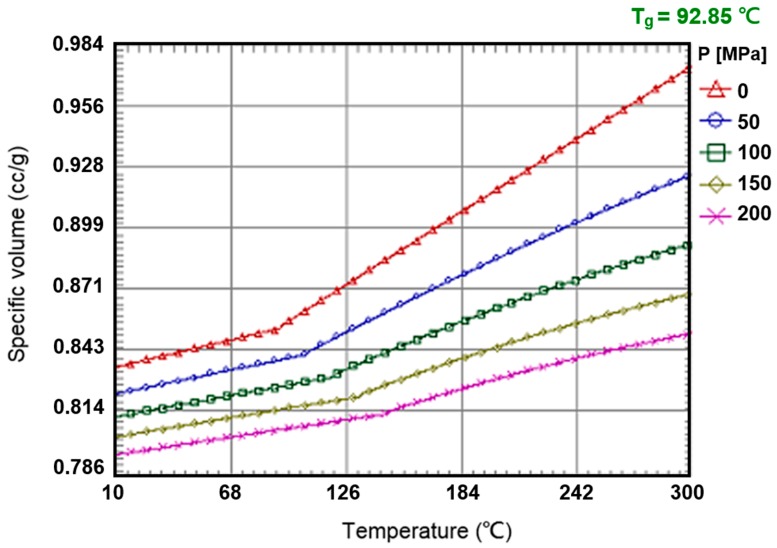
Specific volume curve in Moldex3D of the UV-curable resin.

The UV-curable resin used in the study was an acrylat resin, obtained from Ormocomp (US-S4, Berlin, Germany), with a density of approximately 1.14–1.16 g/cm^3^ and a viscosity of approximately 2000–6000 MPa·s. When the resin’s viscosity increases, the fluid flow is considerably slow, and, therefore, the soft substrate experiences a greater pressure. To make the model more rigorous, we use the upper limit of the viscosity, i.e., 6000 MPa·s, in the simulation. The soft polymer dies were set as a linear elastic material with a Young’s modulus of 41.446 KPa and a Poisson’s ratio of 0.45. The material properties and process conditions are summarized in Table 1, Table 2 and Table 3 and Figure 7, Figure 8, Figure 9 and Figure 10.

### 2.4. Boundary Conditions

**Figure 11:**
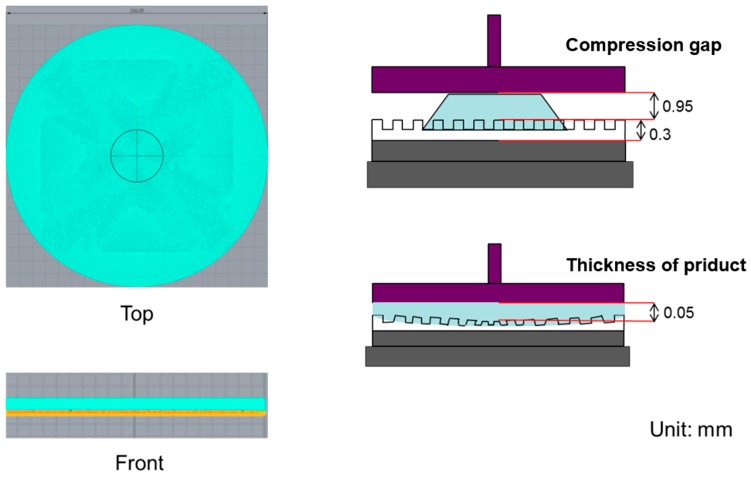
Simulation model established using the Moldex3D software.

The compression simulation conditions were set in the Moldex3D software to be the same as the process conditions summarized in Table 1, including the compression gap, compression time, maximum compression speed, and maximum compression force. When the pressure reaches 90 N, the process will stop until the UV-curable resin has spread and the pressure falls back below the threshold. The compressed gap is the product height. Figure 11 shows a model established using the Moldex3D software.

**Figure 12:**
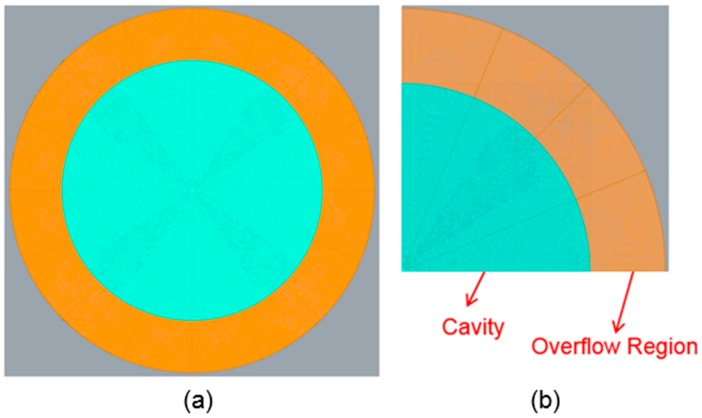
(**a**) Schematic of the overflow region setting in Moldex3D. (**b**) Cavity’s periphery is the overflow area.

To make the UV-curable resin completely form in accordance with the shape of the mold, the boundary condition was set to a fixed end for the general mold–flow analysis. However, in practice, the compression boundary is a free end for releasing the pressure. In other words, after the UV-curable resin has filled into the boundary, it freely overflows from the mold. In this simulation, to mimic these conditions, the overflow area in the periphery is increased such that the material will not be blocked when filled into the boundary. The compression end and the bottom are fixed ends. A schematic of this setup is shown in Figure 12.

**Figure 13:**
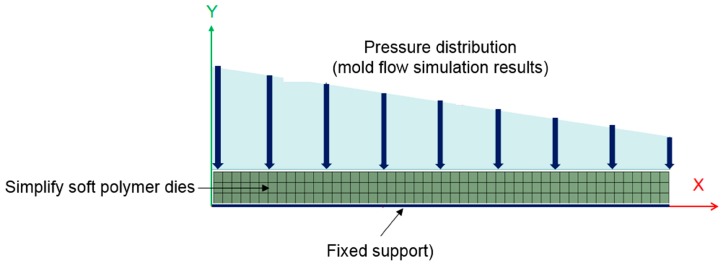
Boundary conditions for the structural analysis (ANSYS).

Figure 13 shows the structural analysis of the model along with the boundary conditions. The soft polymer dies were simplified into a flat plate because the convex features were small and low compared to the size of the model. Because we only aim at observing the non-uniform thickness caused by uneven pressure, we analyze the bottom of the soft polymer dies as a fixed end and assess the pressure distribution at the top of the dies in the mold–flow analysis results as loading.

### 2.5. Mesh Setting

**Figure 14:**
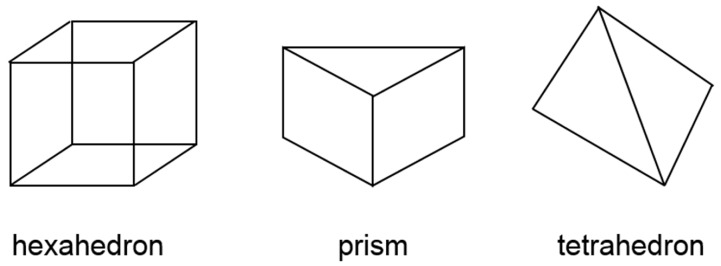
The element shapes for the mesh in the mold–flow simulation (Moldex3D).

A selection of the available mesh shapes is shown in Figure 14. A hexahedron mesh has good convergent. However, in this study, the model is limited by the shape of the disc, so the shape of the mesh was based on the prism element.

**Figure 15:**
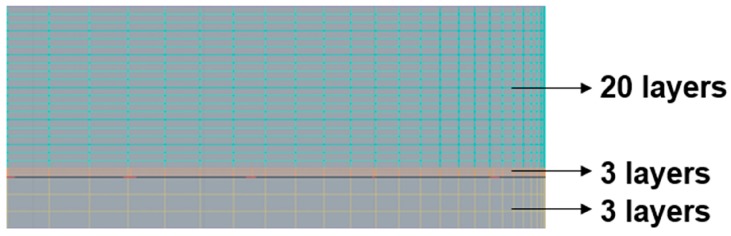
Mesh layers in the compression region (Moldex3D).

With regard to the thickness of the UV-curable resin, if the mesh interval in the compression area is considerably large, the results will have a low resolution. As shown in Figure 15, the soft substrate is divided into three layers, each having a height of 0.3 mm. After compression, the filling compound thickness was 0.05 mm, which was also divided into three layers. The compressed gap was 0.95 mm at the top and was divided into 20 layers.

**Figure 16:**
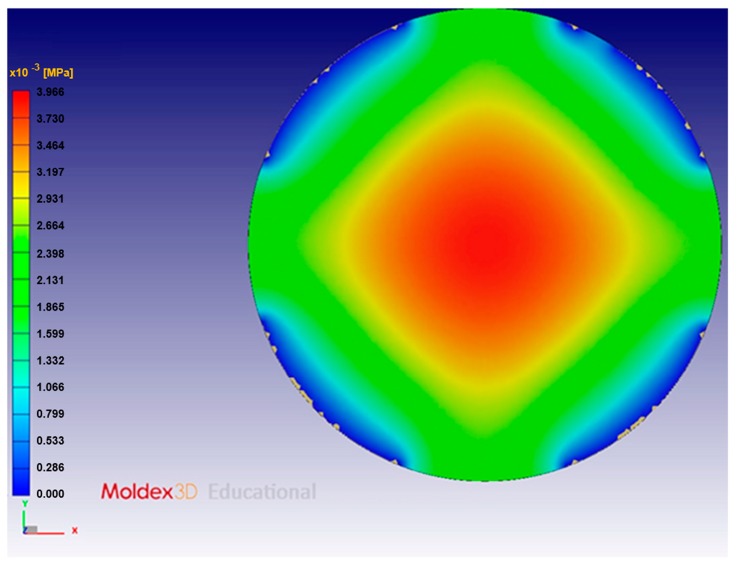
Pressure distribution simulated with a symmetrical coarse mesh: 24,536 triangular elements on the surface (approximately 600,000 elements overall).

**Figure 17:**
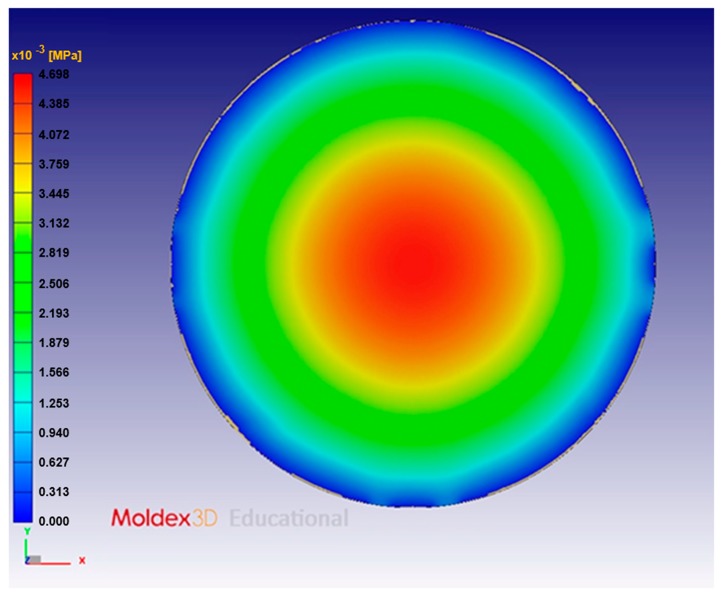
Pressure distribution simulated with a symmetrical fine mesh: 114,452 triangular elements on the surface (approximately 3,000,000 elements overall).

In mesh processing, we also assessed the mesh accuracy. The pressure distributions obtained in the simulation using different meshes are shown in Figure 16 and Figure 17. The results show that the use of a fine mesh and symmetry both improve the resolution of the simulation. The symmetrical fine mesh was therefore used in this study.

## 3. Results and Discussion

### 3.1. Simulation and Measurement Results

**Figure 18:**
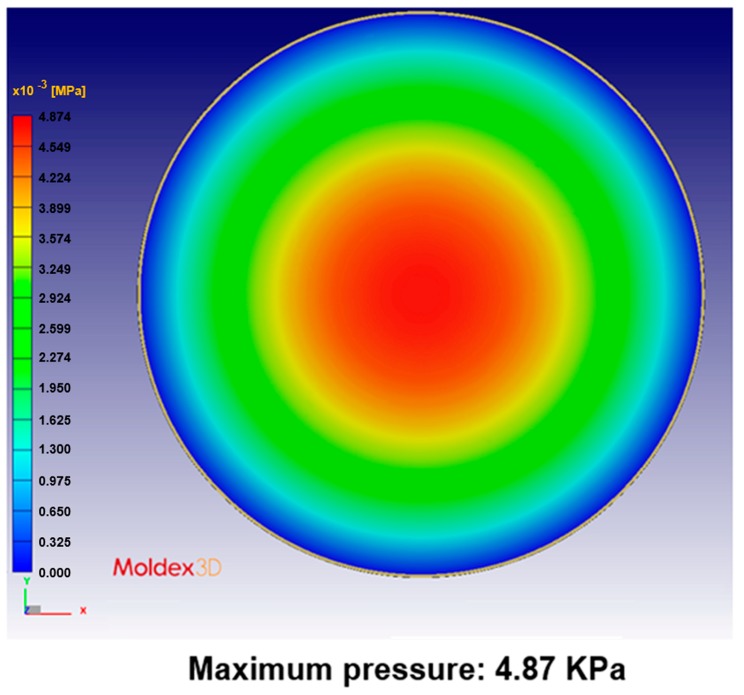
Pressure distribution modeled in a simulation with a single-point gate.

The pressure distribution on the compression mold is the focus of the mold–flow analysis. Figure 18 shows the results of the pressure distribution simulation of compression molding.

**Figure 19:**
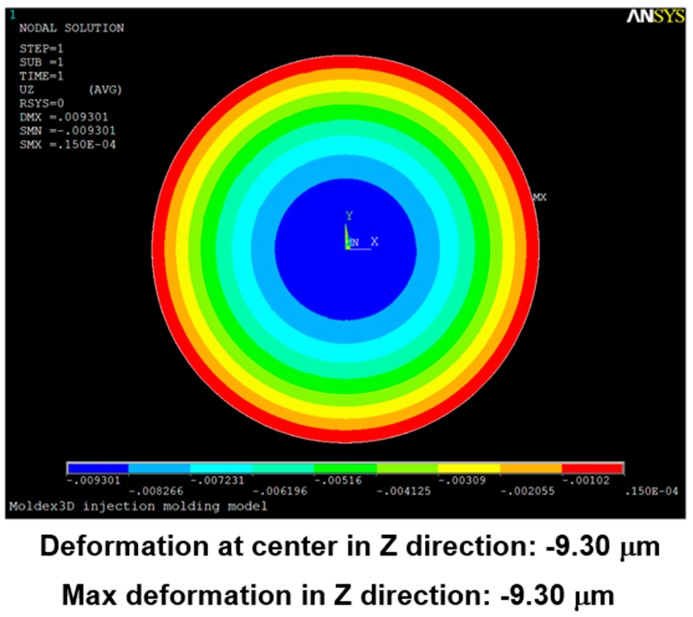
Deformation modeled in a simulation with a single-point gate.

Using the pressure distribution as the load on the soft polymer dies for a structural analysis, the corresponding deformation was obtained, as shown in Figure 19.

The difference in thickness *T_d_* between the center and the edge was approximately 9.3 μm (the maximum deformation at the central position was approximately 9.3 μm, whereas the minimum deformation was close to 0).

**Figure 20:**
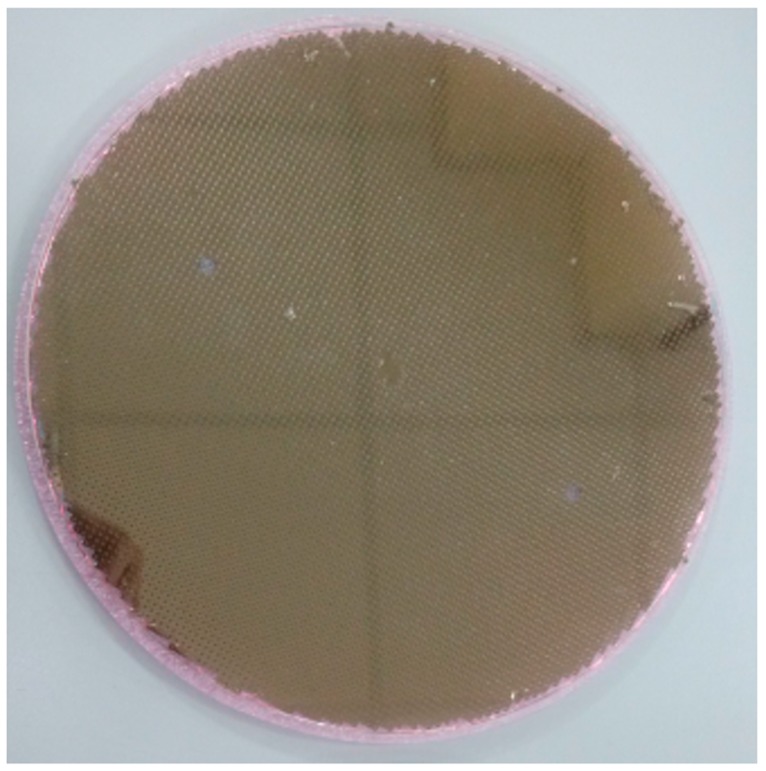
An image of the actual finished product.

An image of the actual finished product is shown in Figure 20. Table 4 summarizes the measurements performed for the product in five independent trials. The average *T_d_* value was approximately 10 μm.

The simulation results were similar to the experimental data. Through this process, the non-uniform thickness resulting from the compression-molding process with a single-point gate was simulated. The error may occur due to the high viscosity setting (range 2000–6000 MPa·s; however, 6000 MPa·s was used in the simulation), the simplicity of the model (convex features were ignored), or the inclusion of an overflow area (the actual situation is free boundary).

### 3.2. Varying the Gate Design

**Figure 21:**
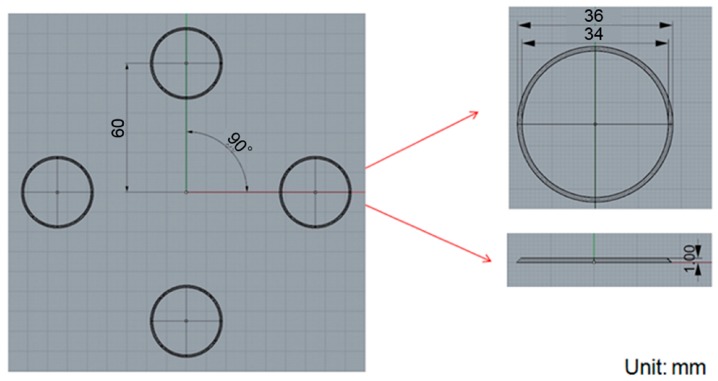
The four-point gate type: the distance between origin and filling shape was about 60 mm, outer diameter and inner diameter are 36 mm and 34 mm, respectively.

**Figure 22:**
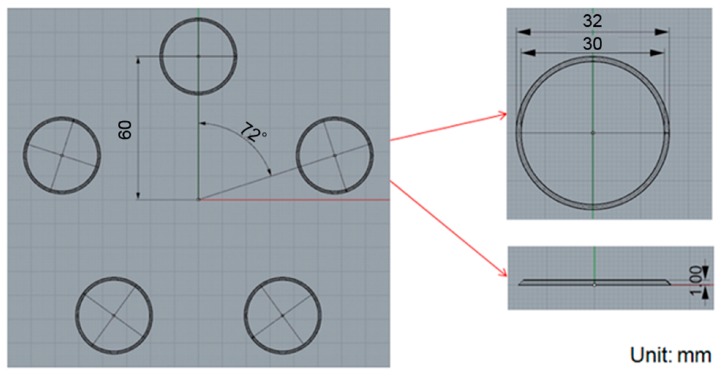
The five-point gate type: the distance between origin and filling shape was about 60 mm, outer diameter and inner diameter are 32 mm and 30 mm, respectively.

**Figure 23:**
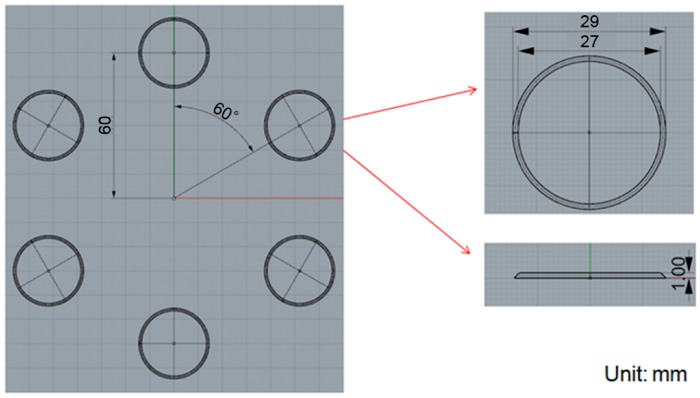
The six-point gate type: the distance between origin and filling shape was about 60 mm, outer diameter and inner diameter are 29 mm and 27 mm, respectively.

We confirmed that the simulation results were consistent with the actual situation and then aimed at improving the original design for this process. To improve the uniformity in the thickness of the product, the type of the gate used in the compression-molding process was changed from a single-point gate to multi-point gates and the effects on the pressure and deformation were characterized. Four-, five- and six-point gates were analyzed, as shown in Figure 21, Figure 22 and Figure 23, respectively.

**Figure 24:**
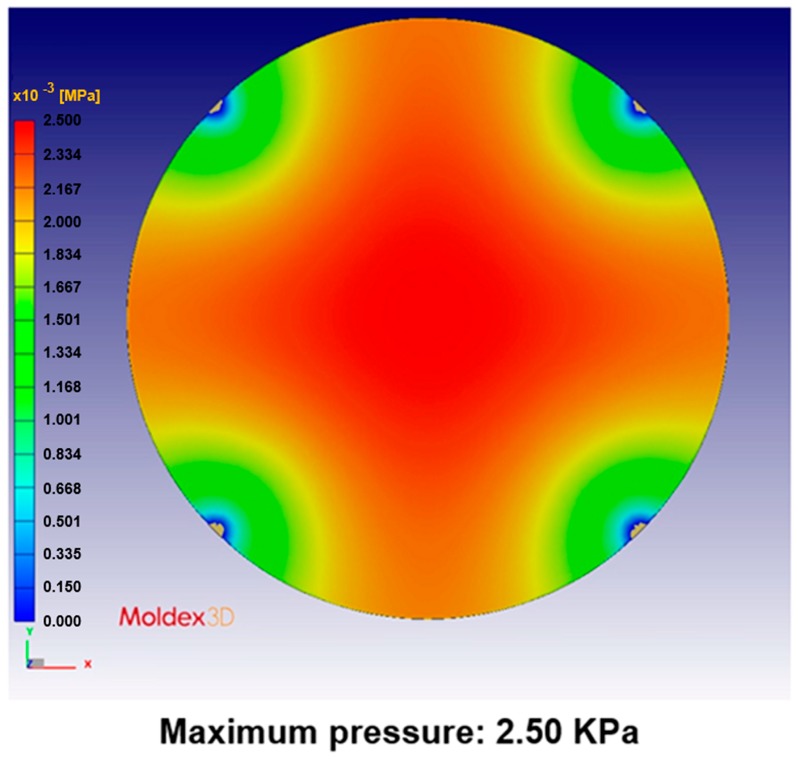
Pressure distribution in the four-point gate simulation.

**Figure 25:**
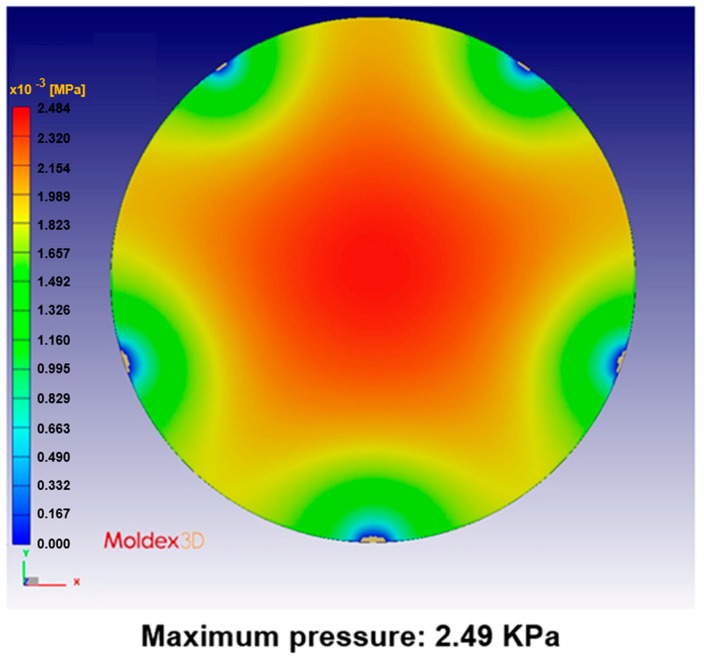
Pressure distribution in the five-point gate simulation.

**Figure 26:**
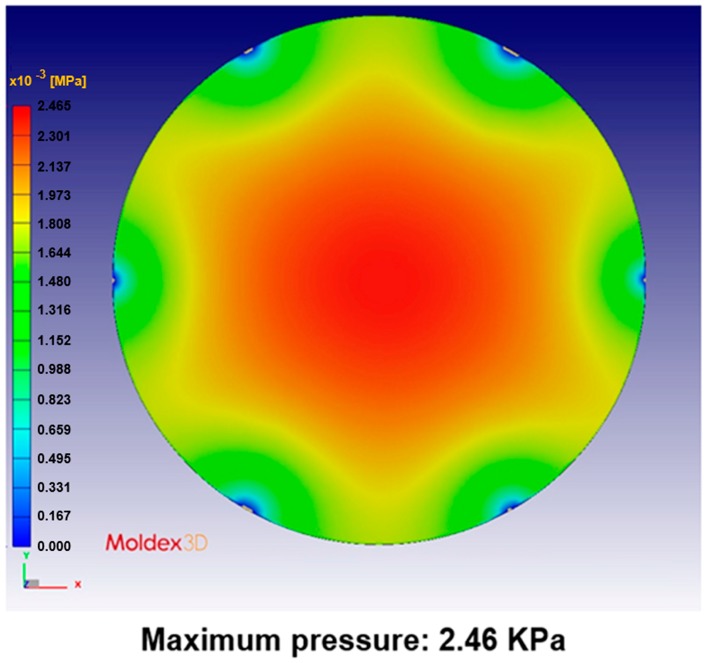
Pressure distribution in the six-point gate simulation.

**Figure 27:**
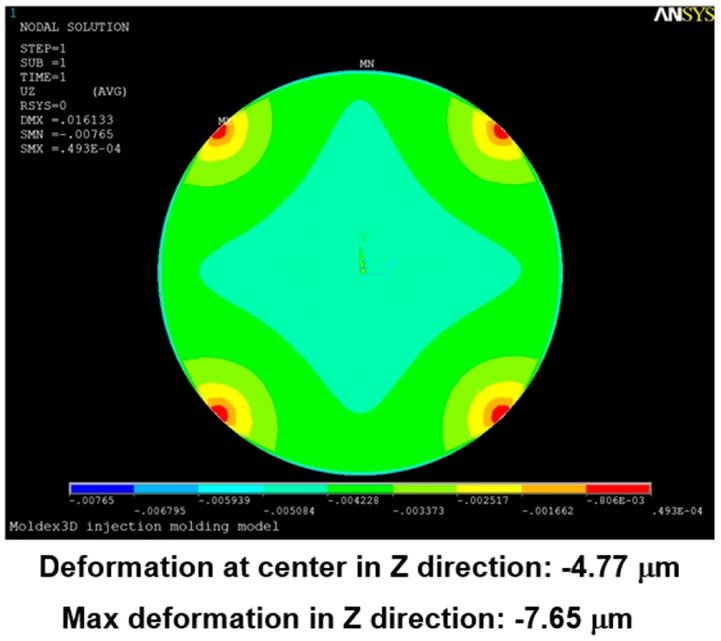
Deformation in the four-point gate simulation.

**Figure 28:**
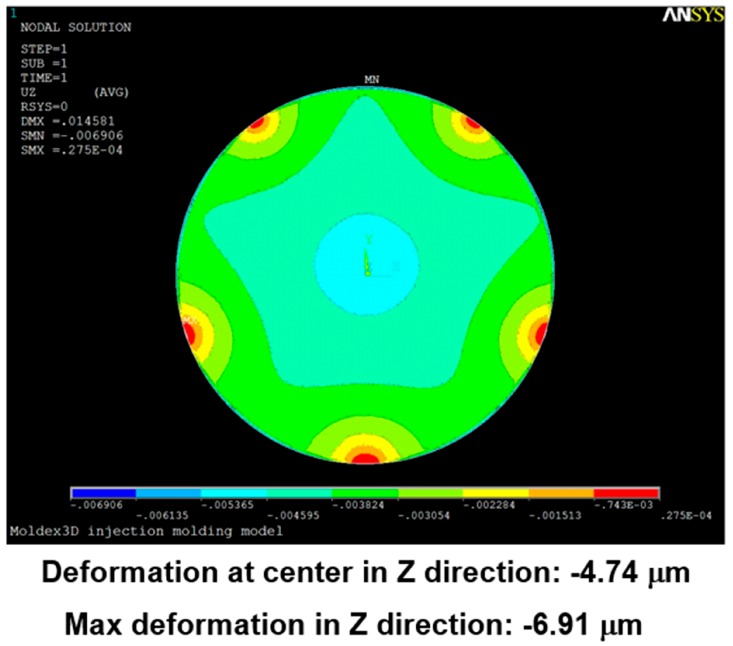
Deformation in the five-point gate simulation.

**Figure 29:**
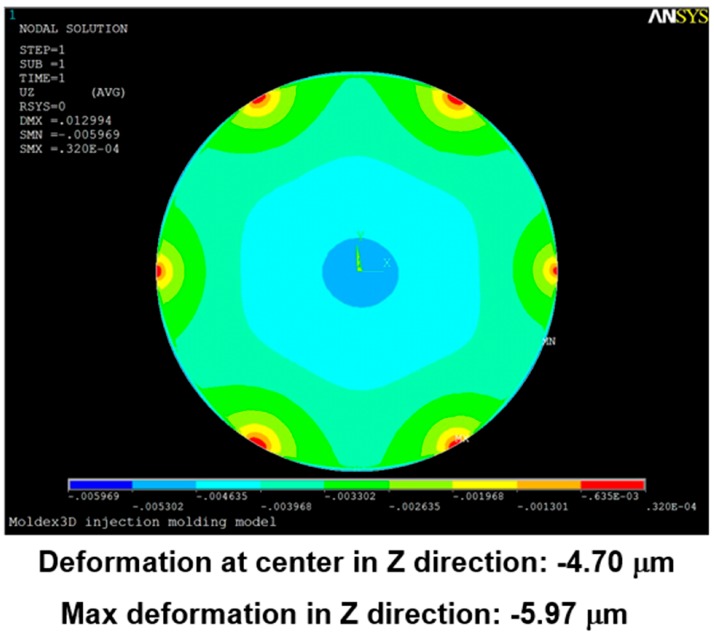
Deformation in the six-point gate simulation.

Figure 24, Figure 25, Figure 26, Figure 27, Figure 28 and Figure 29 show the simulation results. The multi-point gate types can effectively distribute the pressure to avoid it being concentrated in the central position during the compression-molding process.

## 4. Discussion

Based on the previous study, it was known that changing the height of the compression gap or reducing the compression rate could increase the uniformity of the material distribution in the mold. In this study, the compression gap is the thickness of the product, and, therefore, it must be fixed. However, as reducing the compression rate affects the process time, we aimed to change the gate type to improve the uniformity of the product thickness.

In the finite-element simulation, we performed a mold–flow analysis to observe the pressure changes in the mold throughout the process and further combined the results with a structural analysis to predict the non-uniform thickness of the product. The simulation using a single-point gate shows that the pressure is concentrated at the center, causing a significantly non-uniform thickness. The simulation result is consistent with the well-known problem associated with this process and with the experimentally observed results. Hence, the gate type was varied to improve the uniformity of the product.

Table 5 compares the pressure distribution and deformation measured in the mold–flow and structural simulations, respectively, for each gate type. The multi-point gate appears to significantly improve the uniformity in the thickness of the product of the compression-molding process. It is worth noting that in this study, the simulation of the filling boundary was set to have an overflow area, which may allow the peripheral area to experience more pressure than that in the actual situation wherein there is a free boundary that can release the pressure. Therefore, the multi-point gate designs may produce a less non-uniform thickness in the actual situation compared with the simulated result.

## 5. Conclusions

In this study, a feasible simulation is designed for the flow of the UV-curable resin during the compression-molding process. We perform a mold–flow analysis to determine the pressure distribution on the mold and use the results of the analysis to perform a structural analysis for obtaining the deformation (indicating the non-uniform thickness distribution) in the product as a result of the process A.

Using this simulation, it is possible to accurately simulate the non-uniform thickness of the product, which has been widely observed with a single-point gate, demonstrating the feasibility of this study. Subsequently, we simulated different gate types for improving the uniformity in the thickness of the product. The simulation results show that the use of a single-point gate results in high pressure at the center of the mold, whereas the multi-point gates effectively distribute the pressure. This reduces the pressure concentration at the center and effectively improves the uniformity in the thickness of the product.

Furthermore, the problems associated with the compression-molding process have not been comprehensively addressed. The use of a multi-point gate while improving the uniformity in the product’s thickness will inevitably exacerbate the void problem. If a prediction has to be made for the void situation, it is necessary to consider the convex features of the soft polymer dies. This necessitates the use of a significantly higher number of mesh elements, which will load the simulation hardware and software to its limits.

## Figures and Tables

**Table 1:** Process conditions in compression molding.

Parameters of the Compression-Molding Process	
Compression gap	0.95 mm
Compression time	500 s
Maximum compression speed	1 mm/s
Maximum compression force	0.01 tf

**Table 2:** Material properties in the simulation of the mold flow.

Ultraviolet (UV)-Curable Resin	
Density	0.95 mm
Viscosity	600 MPa·s

**Table 3:** Material properties in the simulation of the die structure.

Soft Polymer Dies	
Young’s modulus	41.446 KPa
Poisson’s ratio	0.45

**Table 4:** Measurement data of the product.

*T_d_* (μm)
10.1
10.6
9.8
9.5
10.5
Avg. 10.1

**Table 5:** Pressure distribution and deformation for different gate types.

Simulation Result	Gate Type			
	One Point	Four Points	Five Points	Six Points
Maximum pressure	4.87 KPa	2.50 KPa	2.49 KPa	2.46 KPa
Maximum deformation in the z-direction	−9.30 μm	−7.65 μm	−6.91 μm	−5.97 μm
Deformation at the center in the z-direction	−9.30 μm	−4.77 μm	−4.74 μm	−4.70 μm
